# Antigen Presenting B Cells Facilitate CD4 T Cell Cooperation Resulting in Enhanced Generation of Effector and Memory CD4 T Cells

**DOI:** 10.1371/journal.pone.0077346

**Published:** 2013-10-14

**Authors:** David R. Kroeger, Christopher D. Rudulier, Peter A. Bretscher

**Affiliations:** Department of Microbiology and Immunology, University of Saskatchewan, Saskatoon, Saskatchewan, Canada; Trinity College Dublin, Ireland

## Abstract

We show that the *in vivo* generation of cytokine-producing CD4 T cells specific for a given major histocompatibility class-II (MHCII)-binding peptide of hen egg lysozyme (HEL) is facilitated when mice are immunized with splenic antigen presenting cells (APC) pulsed with this HEL peptide and another peptide that binds a different MHCII molecule. This enhanced generation of peptide-specific effector CD4 T cells requires that the same splenic APC be pulsed with both peptides. Pulsed B cells, but not pulsed dendritic cells (DCs), can mediate CD4 T cell cooperation, which can be blocked by disrupting OX40-OX40L (CD134-CD252) interactions. In addition, the generation of HEL peptide-specific CD4 T cell memory is greater when mice are primed with B cells pulsed with the two peptides than with B cells pulsed with the HEL- peptide alone. Based on our findings, we suggest CD4 T cell cooperation is important for vaccine design, underlies the phenomenon of “epitope-spreading” seen in autoimmunity, and that the efficacy of B cell-depletion in the treatment of human cell-mediated autoimmune disease is due to the abrogation of the interactions between autoimmune CD4 T cells that facilitates their activation.

## Introduction

Cooperation between lymphocytes is essential for the induction of most immune responses. CD4 T cells provide help to B cells to generate antibody-producing cells [1], [2], [3], and to CD8 T cells, through activating an intermediary APC, to produce cytotoxic effector cells [4], [5], [6], [7]. Moreover, antigen can inactivate B cells and CD8 T cells in the absence of helper CD4 T cells [5], [8]. Thus, CD4 T cells generally act as guardians over the fate of B cells and CD8 T cells upon antigen encounter. Knowledge of the circumstances leading to the optimal activation of CD4 T cells is therefore critical to understanding how robust immune responses are generated.

We [9], [10], [11], and others [12], [13], have provided indirect evidence that the optimal activation of CD4 T cells requires lymphocyte cooperation in the form of CD4 T cell collaboration. For example, Gerloni *et al.* reported that mice immunized with a DNA vector encoding a polypeptide generated a greater CD4 T cell response if the vector also encoded an immunodominant peptide recognized by other CD4 T cells [12]. Creusot *et al.* reported that cooperation between two T cell receptor (TCR)-transgenic CD4 T cell populations occurred in mice in response to vaccination with DNA vectors encoding separate polypeptides [13]; cooperation was most efficient when the two vectors were delivered to the same cell. We showed that the *in vitro* generation of delayed type hypersensitivity-mediating cells specific for xenogeneic red blood cells could be helped by CD4 T cells specific for a protein antigen if the protein was chemically linked to the red blood cell. More recently, we showed in BALB/c mice that the *in vivo* generation of CD4 T cells specific for minor peptides of the antigen hen egg lysozyme (HEL) is facilitated by CD4 T cells-specific for the immunodominant peptide, HEL_105–120_ [11].

These observations led to our recent studies that directly demonstrate in BALB/c mice that endogenous subpopulations of CD4 T cells, specific for HEL_105–120_ and for an ovalbumin peptide (OVA_323–339_), can cooperate with one another to increase the number of cytokine-producing CD4 T cells specific for HEL_105–120_ [14]. Immunization with both peptides in IFA generated greater numbers of IL-2, IFNγ, and IL-4 producing CD4 T cells specific for HEL_105–120_ than the numbers generated in mice similarly immunized with HEL_105–120_ alone.

Both DCs and B cells are capable of activating CD4 T cells but, in direct comparisons where antigen presentation is restricted to one cell type, DCs are generally found to be more efficient in carrying out this function [15], [16], [17]. Activation via ligation of CD40 renders tolerogenic resting B cells and DCs potent APC for generating effector CD4 T cells [18], [19], [20]. Given that CD40L (CD154) is present on activated CD4 T cells, it seems plausible that these APC, following antigen-mediated interaction with an activated CD4 T cell, would then be able to potently activate other CD4 T cells. Though both DC and B cells can be activated via ligation of CD40 by CD40L, these APC have different physiological properties, the most obvious of which is the antigen-specific nature of B cells as APC. Differences in antigen processing and their availability within different physiological niches are other properties that set DCs and B cells apart as APC.In order to take our analysis of CD4 T cell cooperation further, we have developed a simple *in vivo* approach where mice are given APC pulsed with one MHCII binding peptide or with this peptide and another that binds to a different host MHCII molecule. We could thus restrict the type of APC presenting the peptide(s), thereby facilitating a level of analysis not achievable by our studies in which peptides were administered in IFA. We assessed the ability of different APC types to support CD4 T cell cooperation, and better characterized the molecular nature of this cooperation. We demonstrate the relevance of CD4 T cell cooperation to the enhanced generation of effector CD4 T cells upon secondary challenge. We discuss our findings in the context of two well-recognized features of autoimmune responses, namely epitope-spreading, and the susceptibility of cell-mediated autoimmune disease to treatment by depletion of B cells.

## Materials and Methods

### Mice

Female BALB/c mice, aged between 6–10 weeks, were obtained from Charles River Canada (Sherbrooke, Canada).

### Animal Ethics

All experiments were conducted under a protocol approved by the University of Saskatchewan’s Animal Research Ethics Board and that adhered to the Canadian Council on Animal Care guidelines for humane animal use.

### Synthetic Peptides

Hen egg lysozyme peptide, HEL_105–120_ (MNAWVAWRNRCKGTDV), and ovalbumin peptide, OVA_323–339_ (ISQAVHAAHAEINEAGR), were synthesized by GenScript (Piscataway, NJ, USA) and were >95% pure, as assessed by mass spectrometry. Peptide antigens were dissolved in PBS, and stored at −80C until needed.

### Peptide Pulsing

In order to load peptide antigens onto splenocyte APC *in vitro*, 1.5–2.5×10^8^ unfractionated splenocytes were cultured overnight in 60 mm tissue culture treated Petri dishes in 3 mL RPMI 1640 medium with penicillin/streptomycin and 2-mercaptoethanol but without serum. These cultures were supplemented with 1 µg/mL LPS from E. coli serotype O111:B4 (Sigma, Saint Louis, MO USA). In appropriate cases splenocytes were cultured in the presence of 50 µM synthetic HEL_105–120_ or OVA_323–339_ peptides.

### Adoptive Transfer of Pulsed Splenocytes

After overnight culture, pulsed splenocytes were washed three times in cold Leibovitz media. In some cases, the pulsed cells were depleted or enriched by MACS before injection. Mice were given antigen-pulsed splenocytes or MACS-purified cells in 25–50 µL cold Leibovitz media subcutaneously in the footpad and lower leg.

### Carboxyfluorescein Succinimidyl Ester (CFSE) Labeling

5×10^7^ cultured splenocytes or freshly isolated DO11.10 splenocytes were labeled in 5 µM CFSE in 1 mL PBS for 5 minutes at room temperature. The cells were then washed three times with ten volumes of PBS to remove any residual dye.

### Magnetic Bead Separations (MACS)

Magnetic labeling and sorting were done according to manufacturer’s recommendations except the buffer (PBS+ 2 mM EDTA) contained no serum. DCs were positively selected by a pan-DC selection Kit, B cells were isolated by negative selection with a B cell selection kit, and DO11.10 CD4 T cells were isolated with a CD4 T cell negative selection kit (Miltenyi Biotech, Auburn, CA USA). All samples were passed over magnetically charged LS columns (Miltenyi Biotech, Auburn, CA USA).

### Enzyme-linked Immunospot (ELISpot) Assay

Analysis of peptide-specific cytokine secretion was accomplished by employing an improved ELISpot assay as previously described [11], [14], [21], [22]. Briefly, a constant number of total leukocytes (1.5×10^6^; normally 10^6^ cells from immunized mice and 5×10^5^ cells from naive mice) were cultured overnight in primary antibody-coated nitrocellulose-bottom wells, of 96-well plates, with and without antigen. After development of cytokine spots, antigen-dependent spots were enumerated by subtracting the numbers of spots counted in wells not supplemented with antigen from the number of spots counted in wells that were supplemented with antigen. For assessing the number of cytokine producing cells in draining lymph nodes, ELISpot wells were supplemented with 10^6^ naive splenocytes and 1/30^th^ of the total lymph node cells were plated per well. Given that the number of cells in the spleen of mice treated differently can vary, all ELISpot data was normalized per spleen or lymph node.

### Immunizations

To generate HEL-specific immune responses, mice were injected intraperitoneally with 200 µL of phosphate buffered saline containing 100 µg heat-aggregated HEL protein.

### Antibodies

The OX40L (CD252) blocking antibody RM134L, and the corresponding rat IgG2b control antibody were obtained, in functional grade from Bio-X-Cell (West Lebanon, NH USA).

### Statistical Analysis

The probability that mean ELISpot responses were not different between two groups was assessed by Student’s T-test. Analysis of variance (ANOVA) was employed to assess whether the mean ELISpot responses differed between multiple groups. Post-hoc analyses employing Bonferroni correction were subsequently employed to compare multiple pairs of means.

## Results

### Immunization with Splenic APC, Pulsed with both HEL_105–120_ and OVA_323–339_, Generates more HEL_105–120_-specifc Cytokine Secreting CD4 T Cells than Immunization with Splenic APC Pulsed with only HEL_105–120_

In order to explore the mechanism whereby CD4 T cells specific for one peptide could help the generation of CD4 T cells specific for another peptide, we developed a system where we could control the type of APC presenting the two peptides. This system involves culturing APC, of various types, with one or more peptides for a period of 12 hours. We included 1 µg/mL LPS in this culture to ensure that the APC were consistently activated to the same degree. We chose to employ HEL_105–120_ and OVA_323–339_ as the “pulsing” peptides as they bind to different MHC molecules, I-E^d^ and I-A^d^ respectively, and therefore do not compete for binding to MHCII molecules.

**Figure 1:**
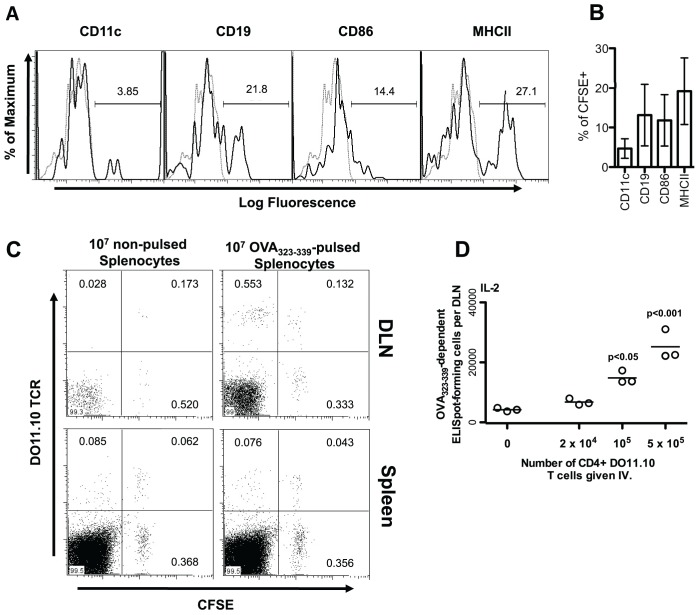
Cultured splenocytes migrate to the popliteal lymph node, present peptide, and activate peptide-specific CD4 T cells, following subcutaneous injection. (A and B) BALB/c splenocytes were cultured overnight in the presence of 1 µg/mL LPS. Cells were harvested and labeled with 5 µM CFSE. After thorough washing, 10^7^ splenocytes were re-suspended and injected subcutaneously into the footpad and lower leg of normal BALB/c mice. After 48 hours, the popliteal lymph nodes of mice were harvested and stained with fluorophore-conjugated antibodies to detect the indicated cell surface proteins. Stained cells were analyzed by flow-cytometry. The expression of individual cell surface proteins is shown for CFSE positive cells. Black histograms show the fluorescence of cells stained with the indicated antibody. Grey, dotted, histograms show the fluorescence of cells stained with a matched fluorophore-conjugated isotype-matched control antibody. (A) Representative histograms are shown. Percentages of stained cells falling within the gated region of the total number of cells within the given plot are shown. (B) Percentages of CFSE+ cells also expressing the indicated marker is shown as the mean +/− standard deviation from individual mice (n = 3). (C) 10^7^ Splenocytes from DO11.10 mice were labeled with 5 µM CFSE, washed, and injected intravenously into BALB/c mice. Twenty-four hours later these mice were injected subcutaneously in the footpad and lower leg with BALB/c splenocytes cultured overnight in the presence or absence of 50 µM OVA_323–339_. Six days post-injection, draining popliteal lymph nodes (DLN) and spleens from injected mice were harvested and stained with a fluorophore-conjugated DO11.10 TCR-specific antibody. The level of CFSE staining and numbers of DO11.10 positive cells were assessed by flow cytometry. Percentages of cells falling within the gate out of the total number of cells within the given plot are shown. (D) One day prior to injection of pulsed splenocytes, BALB/c mice were injected with the indicated number of MACS purified CD4^+^ DO11.10 T cells. BALB/c slpenocytes were cultured overnight in the presence of 50 µM OVA_323–339_. Following harvest and extensive washing, 10^7^ peptide-pulsed splenocytes in 50 µL Leibovitz media were injected into the footpad and lower leg of normal BALB/c mice on days 0, 3, and 6. Nine days after the initial injection of pulsed splenocytes, the OVA_323–339_-specific cytokine producing cells in the draining popliteal lymph node (DLN) were enumerated by ELISpot assay. Each data point represents the number of specific ELISpot-forming cells in the popliteal lymph node of a single mouse. P-values indicate the probability that the number of adoptively transferred DO11.10 CD4 T cells does not increase the number of cytokine producing CD4 T cells generated as assessed by one way ANOVA. *Post-hoc* analysis was employed to compare all groups to that in which mice were given no DO11.10 T cells. The results of a single representative experiment, of two similar experiments, are shown.

Before attempting to generate CD4 T cell responses with the peptide pulsed APC, we assessed whether these APC could migrate to the popliteal lymph node, from the lower leg and footpad, where they could have access to naïve T cells therein. Thus, we transferred 10^7^ non-pulsed CFSE labeled splenocytes into recipient mice 48 hours before harvesting the popliteal lymph node. As shown in Figure 1A and B, approximately 4% of CSFE-labeled cells that migrated to the popliteal lymph node from the lower leg and footpad express the CD11c marker characteristic of conventional DC, while approximately 14% express the B cell marker CD19. About 19% of the CFSE labeled cells present in the popliteal lymph node express MHCII molecules, indicating that these cells were primarily B cells and DCs (14% +4%). We conclude that splenic APC, injected subcutaneously in the footpad and lower leg, are able to traffic to the draining lymph nodes.

We next examined whether transferred splenocytes, pulsed with peptide, could activate CD4 T cells present in the popliteal lymph node. We employed lymphocytes from DO11.10 TCR transgenic mice as the responding CD4 T cells for this purpose. DO11.10 CD4 T cells recognize OVA_323–339_ in the context of I-A^d^. Ten million CFSE labeled DO11.10 spleen cells were injected intravenously into BALB/c mice. Twenty four hours later, 10^7^ BALB/c spleen cells, either pulsed or not pulsed with the OVA_323–339_ peptide, were injected into the footpad and lower leg. Analysis of the CFSE labeled DO11.10 TCR^+^ cells in the popliteal lymph node of these mice, harvested six days after antigen challenge, clearly demonstrated that the administration of OVA_323–339_-pulsed spleen cells caused substantial division of DO11.10 CD4 T cells, while unpulsed APC resulted in only minimal division (80% versus 13% of DO11.10 CD4 T cells were found to be CFSE low; Figure 1C). A similar division of DO11.10 cells was not evident in the spleen.

As shown in Figure 1D, mice given splenocytes pulsed with OVA_323–339_ into the footpad on days 0, 3 and 6 generated only a few cytokine-producing CD4 T cells in the popliteal lymph node by day 9. We hypothesized that cooperative interactions between CD4 T cells would be optimal under conditions where a single peptide-specific population could respond robustly to single peptide-pulsed APC. We thus sought to improve the efficiency of the generation of OVA_323–339_-specific effector cells by seeding mice with DO11.10 CD4 T cells prior to injection. We observed a dose-dependent relationship between the number of DO11.10 CD4 T cells seeded and the number of OVA_323–339_-specific IL-2 producing CD4 T cells generated in the popliteal lymph node (Figure 1D). The lowest number of DO11.10 cells that gave rise to a reliably detectable number of OVA_323–339_ dependent ELISpots was 10^5^. We therefore selected this number of DO11.10 cells as the standard number for our subsequent experiments.

**Figure 2:**
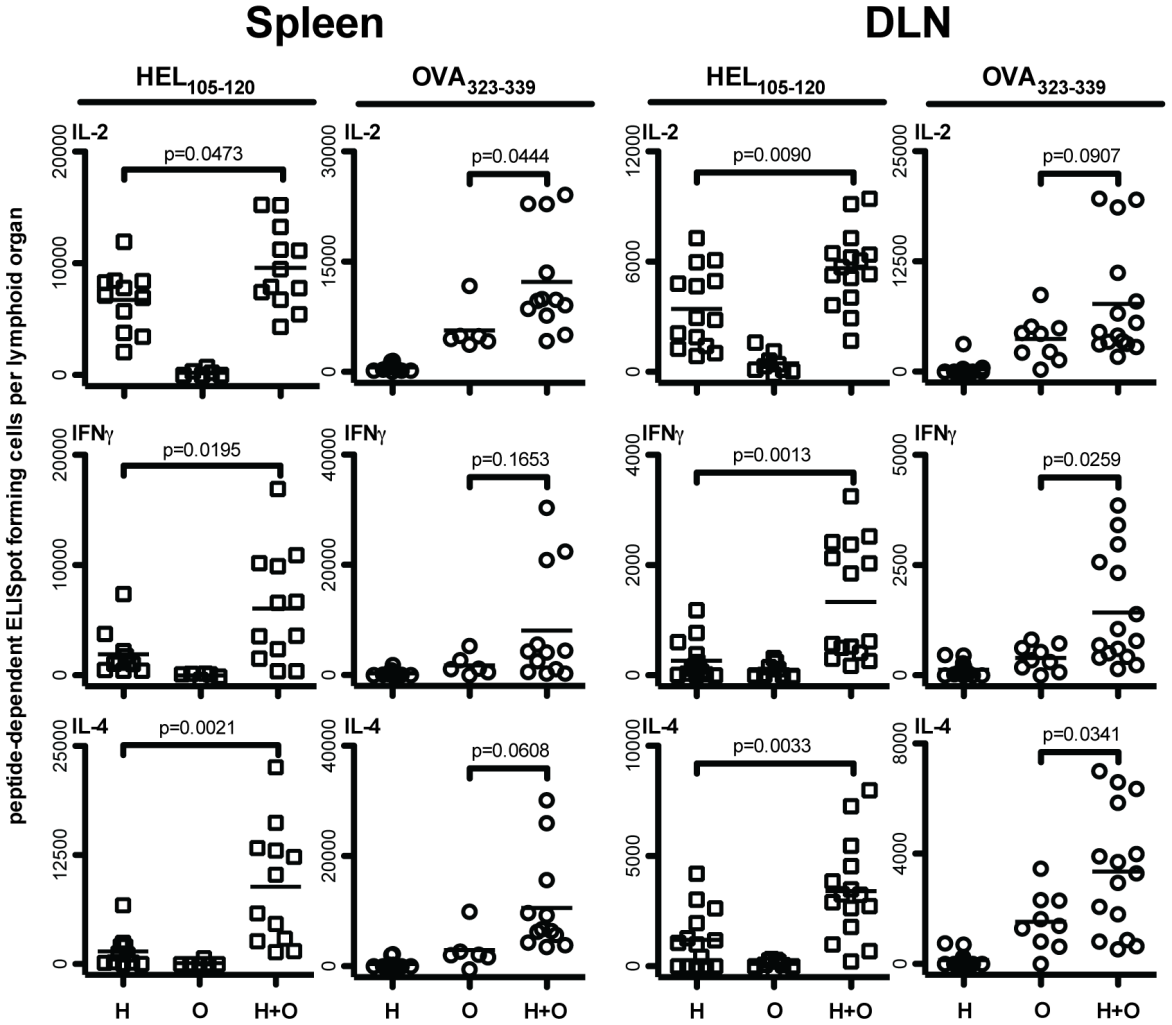
Administration of HEL_105–120_ and OVA_323–339_-pulsed splenocytes to DO11.10-seeded mice results in significant cooperative enhancement of HEL_105–120_-specific effector CD4 T cell generation. One day prior to injection of pulsed splenocytes, BALB/c mice were injected with 10^5^ MACS purified CD4^+^ DO11.10 T cells. BALB/c splenocytes were cultured overnight in the presence of either HEL_105–120_ alone, OVA_323–339_ alone, or both peptides. Following harvest and extensive washing, 10^7^ peptide-pulsed splenocytes were injected subcutaneously into the footpad and lower leg of seeded BALB/c mice on days 0, 3, and 6. Nine days after the initial injection of pulsed splenocytes, the HEL_105–120_ and OVA_323–339_-specific cytokine producing cells in the spleens and draining popliteal lymph nodes (DLN) were enumerated by ELISpot in mice given only HEL_105–120_-pulsed splenocytes (H), OVA_323–339_-pulsed splenocytes (O), and in mice given splenocytes pulsed with both HEL_105–120_ and OVA_323–339_ (H+O). Each data point represents the number of peptide-specific ELISpot-forming cells in the spleen or popliteal lymph node of a single mouse. P-values indicate the probability that the means of the indicated samples are not different as assessed by unpaired, two-tailed T-tests. Results presented are pooled data from four to five independent experiments.

Cooperation between CD4 T cells was directly demonstrated by our finding that the generation of HEL_105–120_-specific IL-2, IFNγ, and IL-4 producing CD4 T cells, in the spleen and draining popliteal lymph nodes, is significantly enhanced in DO11.10-seeded mice given splenocytes pulsed with both HEL_105–120_ and OVA_323–339_ compared with responses in mice given splenocytes pulsed with only HEL_105–120_ (Figure 2). Administration of splenocytes pulsed with OVA_323–339_ alone resulted in the generation of OVA_323–339_- but not HEL_105–120_-specific effector cells, and *vise versa*, indicating that cytokine producing CD4 T cells were activated in a peptide-dependent manner. We also observed a small cooperative effect in the generation of OVA_323–339_-specific IFNγ and IL-4 secreting cells in the draining lymph nodes of mice given splenocytes pulsed with both HEL_105–120_ and OVA_323–339_, which suggests that cooperative responses between CD4 T cells may enhance the activation of all the CD4 T cells involved. However, the presence of OVA_323–339_ on APC helped the generation of cytokine-producing cells specific for HEL_105–120_ more than the presence of the HEL peptide helped the generation of cytokine-producing CD4 T cells specific for OVA_323–339_. This asymmetry may be attributable to the greater than normal number of OVA_323–339_-specific CD4 T cells present in mice that were seeded with 10^5^ DO11.10 cells prior to immunization. We sought to further characterize the effect of CD4 T cell cooperation on the generation of HEL_105–120_-specific CD4 effector cells in this experimental setting.

### Cooperative Enhancement of HEL_105–120_-specific IL-4 Secreting CD4 T Cells, by Co-activation of OVA_323–339_-specific CD4 T Cells, Requires Presentation by the same APC

**Figure 3:**
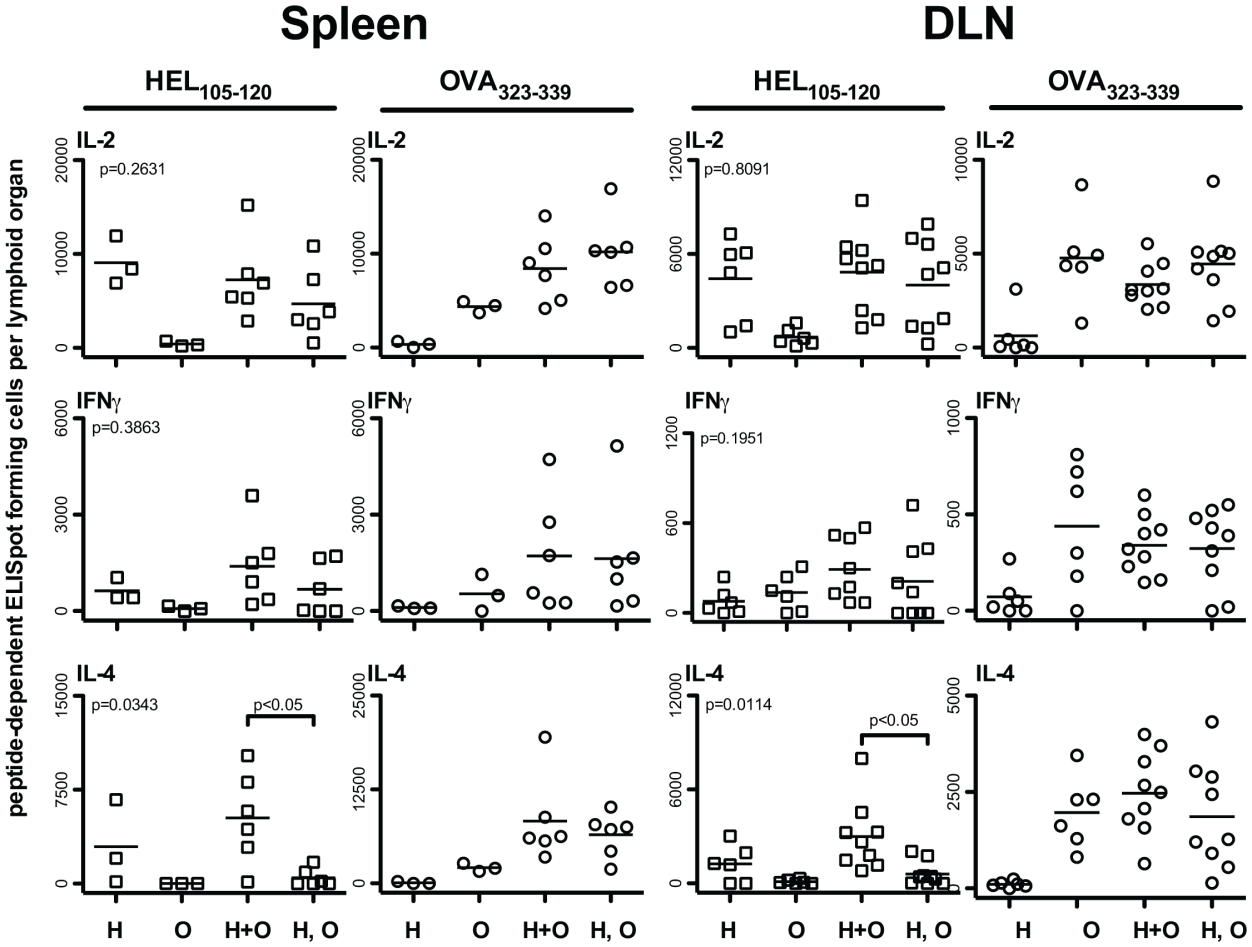
Cooperation between HEL_105–120_ and OVA_323–339_-specific CD4 T cells requires simultaneous presentation of both peptides by the same APC. BALB/c mice were seeded with 10^5^ MACS purified CD4+ DO11.10 T cells one day prior to being injected with 10^7^ splenocytes pulsed with either HEL_105–120_ alone (H), OVA_323–339_ alone (O), both HEL_105–120_ and OVA_323–339_ (H+O), or with 10^7^ H and 10^7^ O splenocytes (H, O). The peptide-specific cytokine producing cells in the spleen and draining lymph nodes of these mice were enumerated by ELISpot on day nine after the first injection. Each data point represents the number of specific ELISpot-forming cells in the lymphoid organ of a single mouse. The probability that the mean number of ELISpot-forming cells between the H, H+O and H, O groups is the same was assessed by one-way ANOVA (P-values; top left corner of all plots). *Post-hoc* comparisons revealed significant differences between the means of the groups indicated. These are pooled results from two to three independent experiments.

An important question regarding CD4 T cell cooperation is whether it generally proceeds via a “linked” mechanism, requiring presentation of two or more peptides on the same APC, or whether two or more T cells, activated simultaneously by different APC, can influence one another through bystander interactions. To address this question, we adoptively transferred splenocytes pulsed with either HEL_105–120_ alone, OVA_323–339_ alone, both HEL_105–120_ and OVA_323–339_, or a mixture of splenocytes separately pulsed with HEL_105–120_ and OVA_323–339_. As can be seen in Figure 3, cooperative enhancement in the generation of HEL_105–120_-specific IL-4 producing CD4 T cells is only achieved when both HEL_105–120_ and OVA_323–339_ are presented on the same APC. We did not observe statistically significant differences in the numbers of HEL_105–120_-specific IL-2, and IFNγ producing cells between mice given splenocytes pulsed with both peptides and those injected with a mixture of singly-pulsed splenocytes. Thus, we conclude that CD4 T cell cooperation for the optimal generation of IL-4 producing cells occurs most efficiently when multiple peptides are presented by the same APC.

### Purified Splenic B Cells Mediate Cooperation between CD4 T Cells Specific for HEL_105–120_ and OVA_323–339_

The nature of the APC that mediates CD4 T cell cooperation has implications for the physiological role that this process may play in the generation of immune responses. We found that pulsed B cells and DC reached the draining lymph node after injection (Figure 1A, B), and therefore we sought to determine whether either of these cell types could mediate CD4 T cell cooperation.

**Figure 4:**
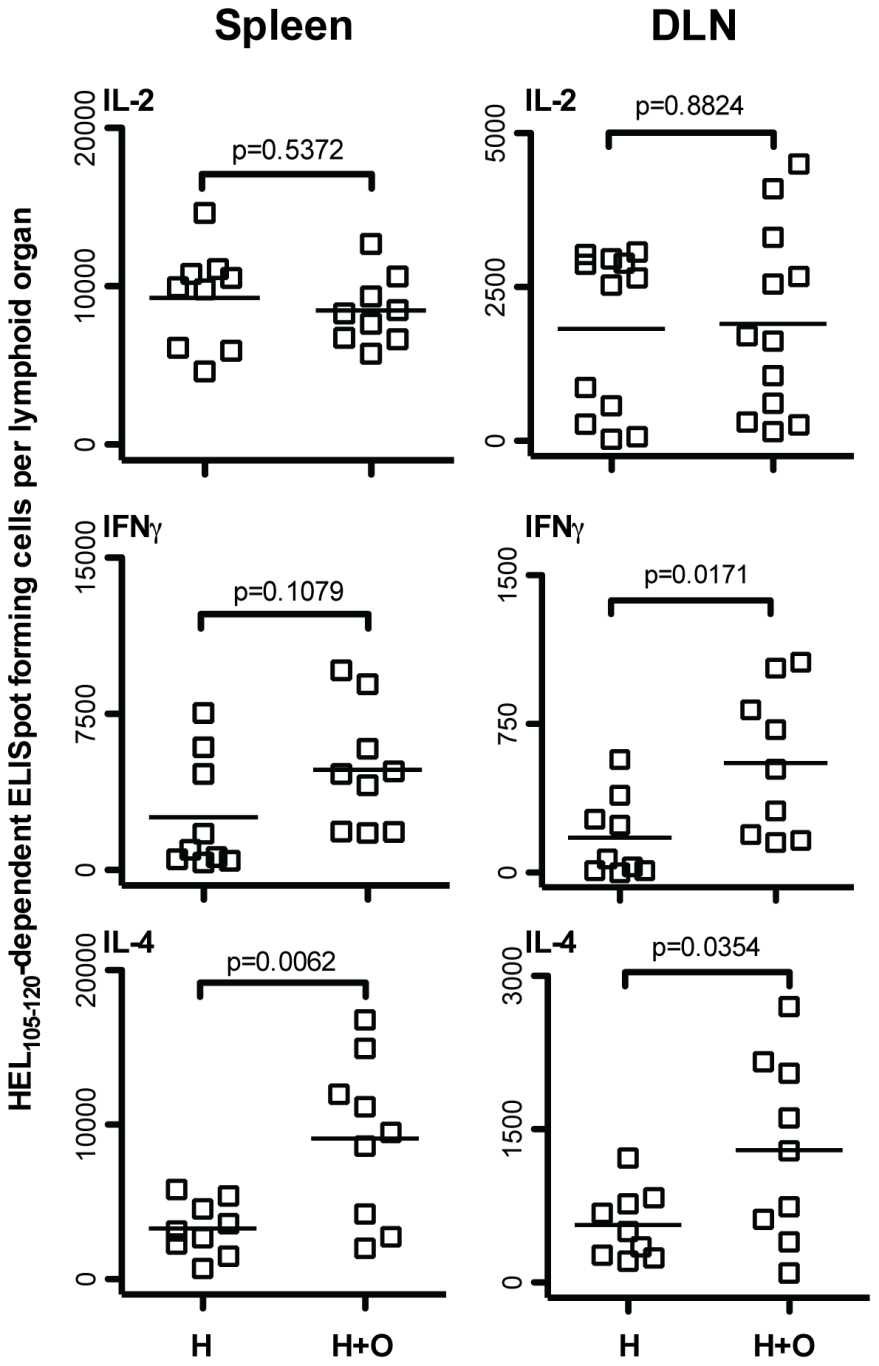
Purified B cells mediate cooperation between HEL_105–120_ and OVA_323–339_-specific CD4 T cells. BALB/c mice were injected with 10^5^ MACS purified CD4+ DO11.10 T cells one day prior to injection. Splenocytes from BALB/c mice were cultured in the presence of HEL_105–120_ alone or both HEL_105–120_ and OVA_323–339_. After harvesting and washing, the splenic B cells were isolated by negative selection. B cells were injected in proportion to the standard injection of 10^7^, 3.5×10^6^ per mouse. On day 9 after the first injection the HEL_105–12_-specific cytokine-producing cells in the spleens, and draining popliteal lymph nodes (DLN), were enumerated by ELISpot, in mice given HEL_105–120_-pulsed B cells (H), or HEL_105–120_ and OVA_323–339_-pulsed B cells (H+O). Each data point represents the number of specific ELISpot-forming cells in the lymphoid organ of a single mouse. P-values indicate the probability that the means of the indicated samples are not different as assessed by unpaired, two-tailed T-tests. Data presented are pooled values from three independent experiments.

We pulsed whole splenocytes with a single peptide or with both HEL_105–120_ and OVA_323–339_ and then isolated B cells, by negative selection over magnetic columns, prior to adoptive transfer. Our isolation protocol resulted in untouched B cells that were at least 95% pure and expressed both MHCII and CD86 (not shown). Cell recovery following B cell isolation was generally 35–40% of the pulsed splenocytes and mice were therefore immunized with 3.5×10^6^ purified B cells. With this approach, we found that purified pulsed B cells presenting both HEL_105–120_ and OVA_323–339_ facilitated the enhanced generation of HEL_105–120_-specific IL-4 and IFNγ producing CD4 T cells (Figure 4). We observed either no effect, or a weak cooperative effect, consistent with observations shown in previous figures, in the generation of OVA_323–339_-specific effector cells when mice were immunized with B cells pulsed with OVA_323–339_ or HEL_105–120_ and OVA_323–339_ (not shown). Thus, the adoptive transfer of highly purified peptide-pulsed B cells leads to a faithful recapitulation of our findings made with unfractionated splenocytes. We conclude that B cells have the capacity to mediate CD4 T cell cooperation.

### Enriched Splenic DCs do not Appear to Mediate Cooperation between CD4 T Cells

**Figure 5:**
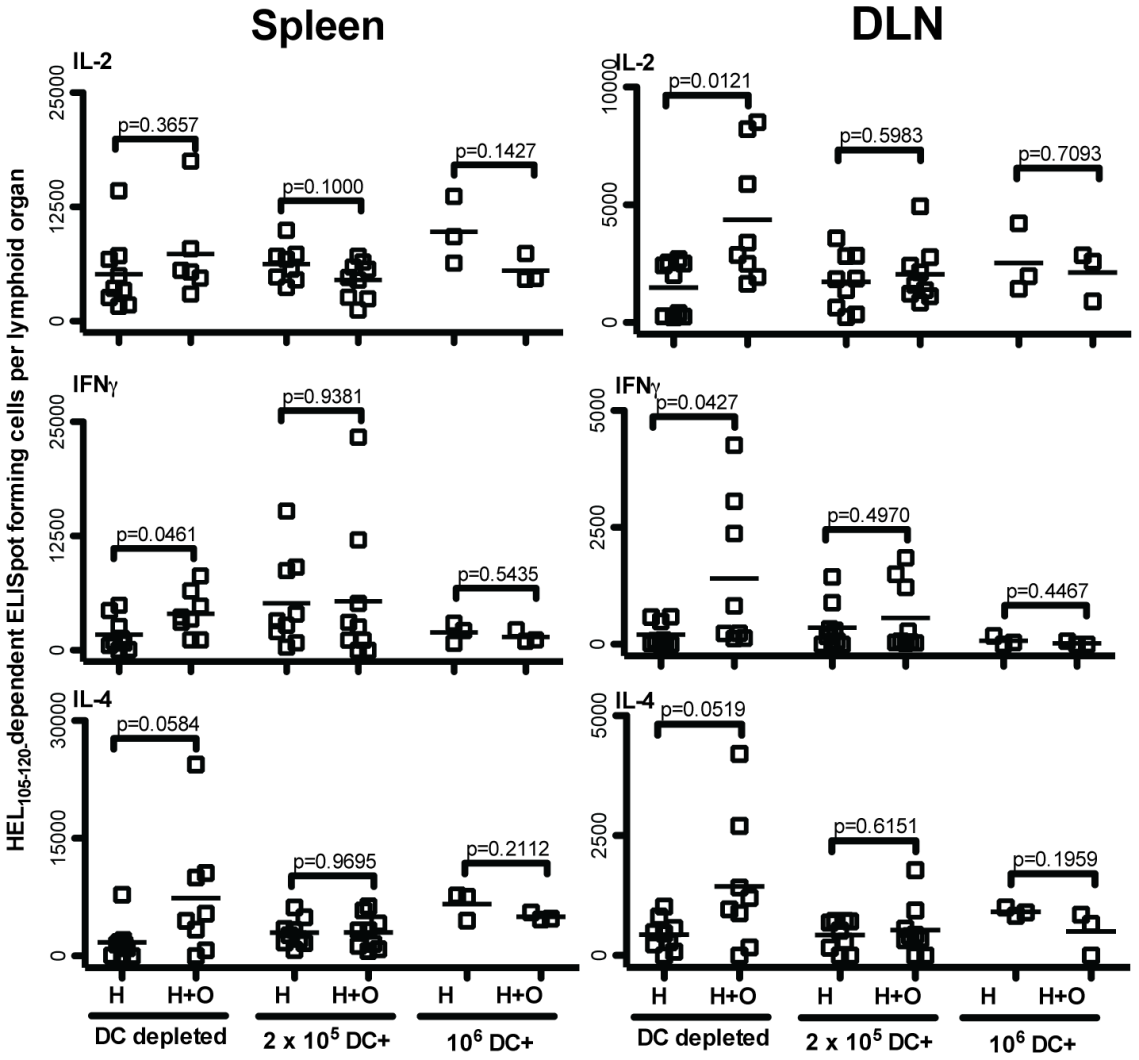
Enriched splenic DCs do not mediate cooperation between HEL_105–120_ and OVA_323–339_-specific CD4 T cell populations. BALB/c mice were seeded with 10^5^ MACS purified CD4^+^ DO11.10 T cells one day prior to injecting splenocytes cultured in the presence of HEL_105–120_ alone or in the presence of both HEL_105–120_ and OVA_323–339_. After harvesting and washing, splenic DC populations were isolated by positive selection. Both DC depleted and DC enriched (DC+) peptide-pulsed cells were injected in proportion to the standard injection of 10^7^ (10^7^ and 2×10^5^ cells respectively). In one experiment the number of DC enriched cells was increased five-fold (10^6^ DC+). These injections were given on days 0, 3, and 6. On day 9 after the first injection, the HEL_105–120_-specific cytokine-producing cells in the spleens, and draining popliteal lymph nodes (DLN), were enumerated by ELISpot, in mice given HEL_105–120_-pulsed APC (H), or HEL_105–120_ and OVA_323–339_-pulsed APC (H+O). Each data point represents the number of specific ELISpot-forming cells in the lymphoid organ of a single mouse. P-values indicate the probability that the means of the indicated samples are not different as assessed by unpaired, two-tailed T-tests. The data presented are pooled values from three independent experiments.

Given that splenic DCs, consisting of both plasmacytoid and conventional DCs, are capable of potently activating CD4 T cells, we explored whether these cells could also mediate cooperation between CD4 T cells. We positively enriched total splenic DCs from pulsed spleen cells prior to adoptive transfer, resulting in a yield of 2% (2×10^5^). At least 70% of the cells isolated via this protocol expressed MHCII and resembled activated DCs in their expression of CD11c and CD86, while the remaining cells in our preparation displayed characteristics of dead or dying cells, which are well known to non-specifically bind magnetic beads (not shown). Although peptide-pulsed DC-enriched cells were potent activators of CD4 T cells, resulting in about 5000 HEL-_105–120-_specific IL-2 producing cells per spleen in mice immunized with 2×10^5^ HEL_105–120_-peptide-pulsed DC, these DC did not mediate cooperative enhancement of the generation of HEL_105–120_-specific cytokine producing CD4 T cells. Even when the number of transferred DCs was increased from 2×10^5^ to 10^6^, no effect of CD4 T cell cooperation was observed (Figure 5). We conclude that splenic DCs do not efficiently facilitate cooperation between CD4 T cells.

### Activation of CD4 T Cells, by Peptide-presenting Purified B Cells, is Inhibited by BlockingOX40-OX40L Interactions

**Figure 6:**
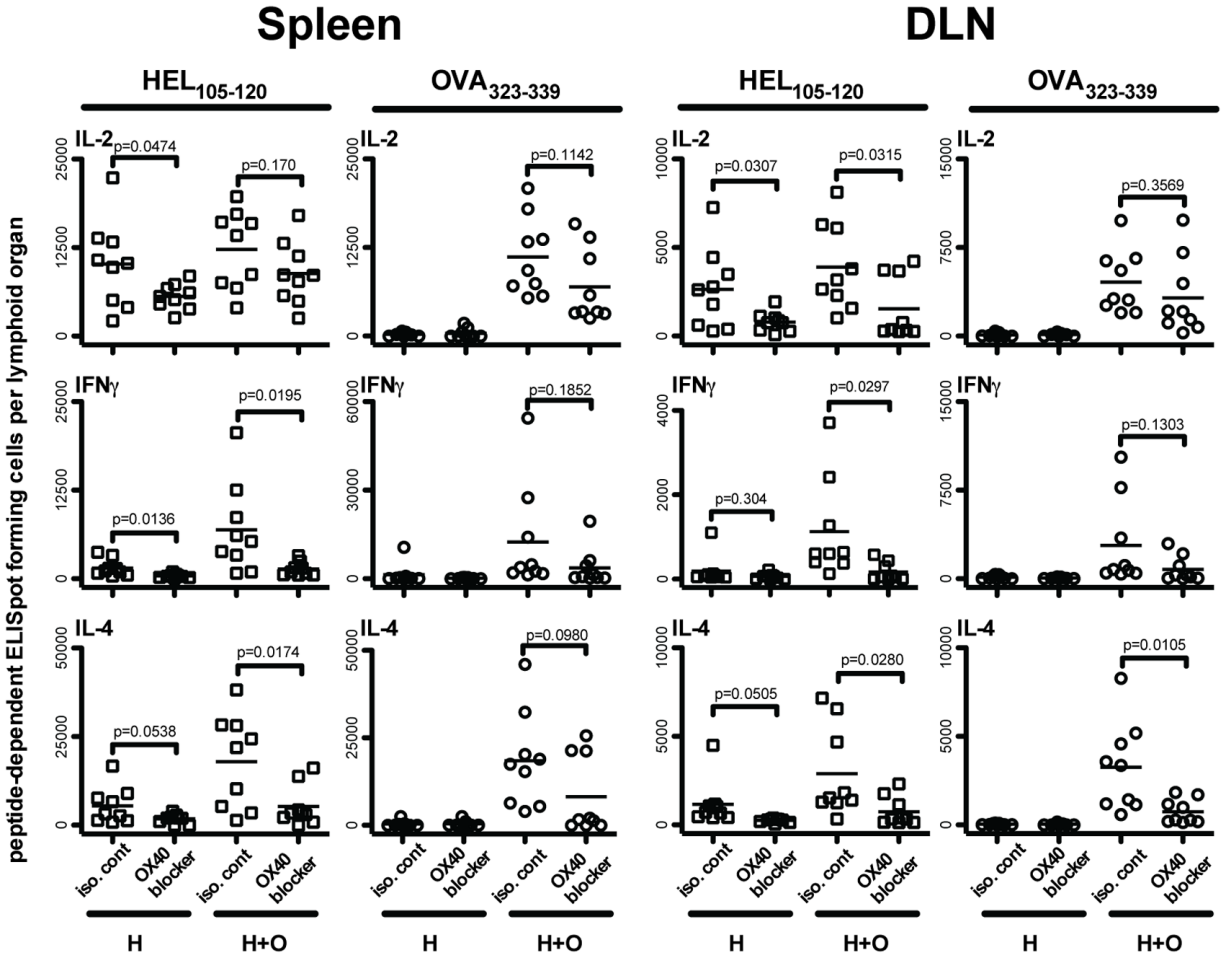
OX40L blockade significantly impairs the generation of peptide-specific cytokine producing effector CD4 T cells following injection of peptide-pulsed purified B cells. One day prior to injection of pulsed B cells, BALB/c mice were injected with 10^5^ MACS purified CD4^+^ DO11.10 T cells. BALB/c splenocytes were cultured overnight in the presence of HEL_105–120_ alone, or both HEL_105–120_ and OVA_323–339_. Following splenocyte harvest, B cells were isolated by MACS negative selection and 3.5×10^6^ peptide-pulsed B cells were injected subcutaneously into the footpad and lower leg of DO11.10-seeded BALB/c mice on days 0, 3, and 6. With each B cell injection, mice were injected intravenously with either 50 µg of an isotype-matched control antibody (iso. cont.) or with an OX40 blocking antibody (OX40 blocker). Nine days after the initial injection of pulsed splenocytes, the HEL_105–120_ and OVA_323–339_-specific cytokine producing cells in the spleens and draining popliteal lymph nodes (DLN) were enumerated by ELISpot for mice given only HEL_105–120_-pulsed B cells (H), and in mice injected with B cells pulsed with both HEL_105–120_ and OVA_323–339_ (H+O). Each data point represents the number of specific ELISpot-forming cells in the spleen and popliteal lymph node of a single mouse. P-values indicate the probability that the means of the indicated samples are not different as assessed by unpaired, two-tailed T-tests. Data presented are pooled values from three independent experiments.

In our recent study [14], we observed that OX40-OX40L (CD134–CD252) interactions are involved in CD4 T cell cooperation leading to the enhanced generation of cytokine-producing CD4 T cells. Similar observations have been reported by Gerloni *et al.* [12]. Given these findings, we examined the role of OX40-OX40L in B cell-mediated CD4 T cell cooperation, by administering non-depleting antagonistic anti-OX40L antibody, in a manner employed by others to block OX40-OX40L interactions [23], [24], [25], to mice seeded with DO11.10 CD4 T cells and immunized with purified B cells pulsed with HEL_105–120_ alone or with both HEL_105–120_ and OVA_323–339_ together. Treatment with anti-OX40L antibody, when compared to treatment with the isotype-matched control antibody, dramatically reduced the number of HEL_105–120_ specific cytokine producing CD4 T cells generated, regardless of whether the mice were immunized with B cells that were pulsed with HEL_105–120_ alone or with HEL_105–120_ and OVA_323–339_ together (Figure 6). Blocking OX40L also had a negative effect on the generation of OVA_323–339_ specific effector cells as seen in the spleen and a smaller effect as seen in the draining lymph node. Thus, it appears that the availability of OX40L during priming of CD4 T cells by B cells alone is necessary for the generation of optimal numbers of effector CD4 T cells. This finding is consistent with the observations of others [26], and with the hypothesis that an important role of the B cell, in CD4 T cell priming, is in facilitating cooperation between CD4 T cells and that this process involves OX40-OX40L interactions.

### Cooperation of CD4 T Cells Leads to Enhanced Secondary Effector CD4 T Cells Generated upon a Subsequent Challenge

**Figure 7:**
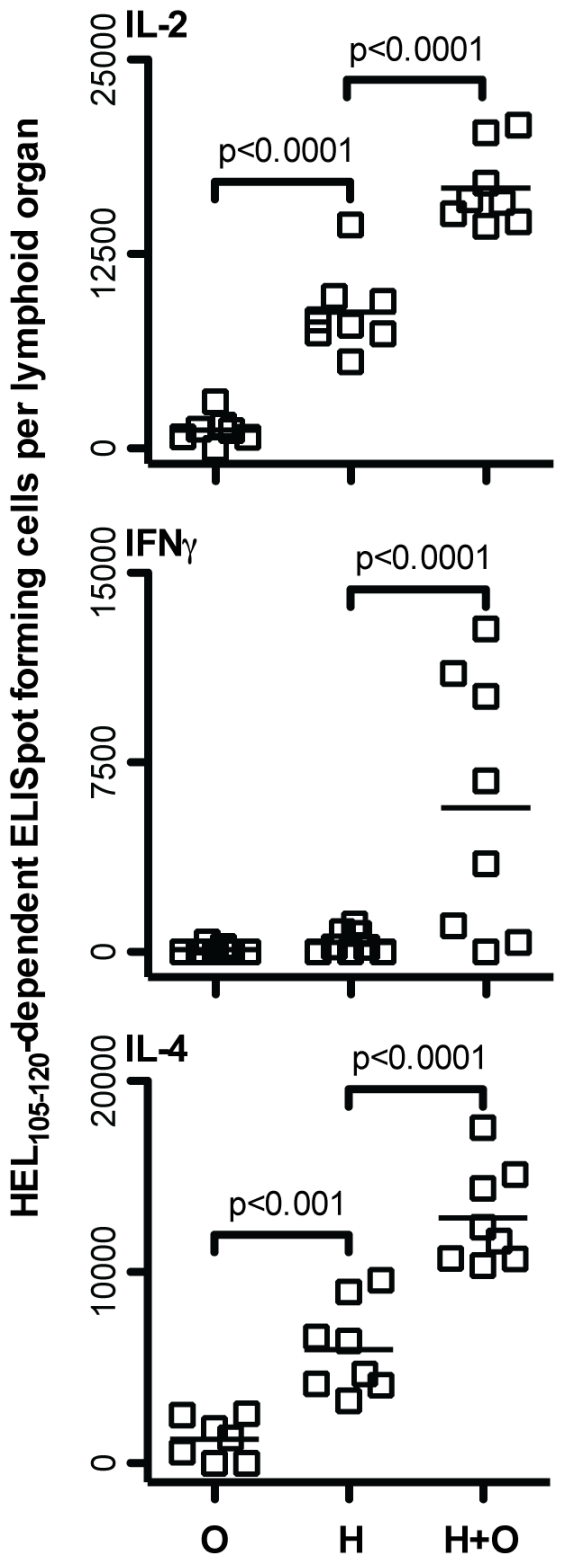
Cooperative enhancement of HEL_105–120_-specific effector CD4 T cell generation results in increased HEL_105–120_-specific effector cell responses following secondary immunization. One day prior to injection of pulsed splenocytes, BALB/c mice were seeded with 10^5^ MACS purified CD4^+^ DO11.10 T cells. BALB/c splenocytes were cultured overnight in the presence of either OVA_323–339_ alone, HEL_105–120_ alone, or both HEL_105–120_ and OVA_323–339_. Following harvest, B cells were isolated by MACS negative selection and 3.5×10^6^ peptide-pulsed B cells were injected subcutaneously into the footpad and lower leg of seeded BALB/c mice on days 0, 3, and 6. Seven weeks later all mice were injected intraperitoneally with 100 ug heat-aggregated HEL protein in saline. Ten days post-challenge the HEL_105–120_-specific cytokine producing cells in the spleen of mice given OVA_323–339_-pulsed B cells (O), HEL_105–120_-pulsed B cells (H), and OVA_323–339_ and HEL_105–120_-pulsed B cells (H+O) were assessed by ELISpot. Each data point represents the number of specific ELISpot-forming cells in the spleen of a single mouse. The probability that the mean number of ELISpot-forming cells in all groups is the same was assessed by one-way ANOVA. Post-hoc comparisons revealed significant differences between the means of the groups indicated. These are pooled results of three independent experiments.

Given that CD4 T cell cooperation amongst HEL_105–120_ and OVA_323–339_ specific CD4 T cells increased the number of HEL_105–120_-specific T cells in a primary response, and that the magnitude of a secondary CD4 T cell response is often proportional to the number of effectors generated in a primary response, we wished to investigate whether cooperation between these T cells could also increase recall CD4 T cell responses to HEL_105–120_. To test this possibility, we immunized mice, seeded with DO11.10 CD4 T cells, with purified B cells pulsed with only OVA_323–339_, only HEL_105–120_, or pulsed with both peptides. Some mice were sacrificed at day 9 and their spleen and draining lymph node assessed for HEL_105–120_-specific and OVA_323–339_-specific effector CD4 T cells and the anticipated responses were found, as seen in Figure 3. After resting the remaining mice for seven weeks, they were challenged intraperitoneally with 100 µg heat-aggregated HEL protein in saline. Mice that were given B cells pulsed with HEL_105–120_ alone had significantly higher numbers of HEL_105–120_-specific IL-2 and IL-4 producing cells in the spleen on day 10 after challenge than did mice given control B cells treated with OVA_323–339_ alone (Figure 7). Significantly, mice that received B cells pulsed with HEL_105–120_ and OVA_323–339_ together during the priming phase had even greater numbers of HEL_105–120_ specific effector cells in the spleen following secondary immunization than did those given B cells pulsed with HEL_105–120_ alone. Thus, it appears that cooperation between CD4 T cell subpopulations, mediated by B cells during the priming phase, results in enhanced CD4 T cell recall responses upon challenge. We conclude that CD4 T cell cooperation can have a positive effect on the generation of CD4 T cell memory.

## Discussion

Cooperation between CD4 T cells, leading to their enhanced activation, has been reported previously. However, due to outstanding questions regarding the cellular and molecular mechanisms by which CD4 T cell cooperation occurs, the physiological relevance of this process has not been well recognized. We show that antigen-pulsed B cells, but not splenic DCs, can mediate cooperative enhancement of the activation of CD4 T cells and that there is a critical role for OX40/OX40L during activation of CD4 T cells by peptide-pulsed B cells. Moreover, B cell mediated CD4 T cell cooperation led to enhanced memory at the level of CD4 T cells.

We would like to address particular aspects of our findings before discussing their general significance. Firstly, our finding, that both the peptides have to be on the same APC for this APC to mediate CD4 T cell cooperation, leads us to infer that the loss of peptide from the administered APC, and the subsequent binding of the disassociated peptide to host APC, does not play a significant role in the CD4 T cell cooperation we observe. Secondly, the facilitation we observe of HEL_105–120_-specific CD4 T cell activation by OVA_323–339_-specific CD4 T cells is much more evident than the facilitation of the activation of OVA_323–339_-specific CD4 T cells by HEL_105–120_-specific CD4 T cells. We think this is explained by the fact that we chose to administer a number of OVA_323–339_-peptide specific transgenic CD4 T cells that, by themselves, resulted in the readily detectable activation of the OVA_323–339_-specific CD4 T cells by APC pulsed with the OVA_323–339_ alone. This number of transgenic OVA_323–339_-specific CD4 T cells is most likely considerably larger than the number of endogenous HEL_105–120_-specific CD4 T cells present. We therefore expect the activation of the HEL_105–120_-specific CD4 T cells by APC pulsed with the HEL_105–120_ alone to be more limited by the scarcity of CD4 T cells than the activation of OVA_323–339_-specific CD4 T cells by OVA_323–339_-pulsed APC. Thirdly, the facilitation of CD4 T cell activation is overall most apparent in the generation of IL-4-producing CD4 T cells than in the generation of IFNγ- or IL-2-producing CD4 T cells. Other studies [27], [28], [29], [30] show that the *in vivo* generation of IL-4 producing cells is more dependent on the number of antigen-specific CD4 T cells present than is the generation of IFNγ producing CD4 T cells, a finding that is in line with our current observations. We cannot deduce from the observations reported here the degree to which the generation of cytokine-producing cells, obtained by administering APC pulsed with just one peptide, involves CD4 T cell cooperation. Our previous *in vivo* observations appear to show that CD4 T cell cooperation facilitates the generation of HEL-specific IL-2-, IFNγ- and IL-4-producing CD4 T cells [11], [31]. Lastly, some might consider that the CD4 T cell responses we detect are not very large. We should point out in this context that our previous studies show that immunization with HEL, in two different inbred strains of mice, results in the generation of CD4 T cells specific for about six non-overlapping peptides [11]. We chose in this study to use only two peptides, and two that bound to different host MHC molecules, to avoid the complexities of any competition between peptides binding to the same MHC molecules during pulsing. However, if several peptides are naturally generated through processing of the immunizing antigen and presented by APC, and if CD4 T cells specific for all these different peptides can mutually facilitate each other’s activation, CD4 T cell cooperation could be a very effective means of facilitating the generation of activated CD4 T cells. Thus, the observations made with two peptides might be much more dramatic in natural situations where more peptides are generated and presented. We feel these considerations are particularly significant in the context of our finding that CD4 T cell cooperation facilitates the generation of CD4 T cell memory, and in the context of vaccine design.

We hypothesize that CD4 T cell cooperation underlies the phenomenon of epitope spreading. Epitope spreading is a process whereby the number of B cell- and T cell-epitopes responded to increases over the course of the immune response. This phenomenon is evident during infections [32], and after experimental immunization, but is most commonly discussed in terms of the development of autoimmunity [33], [34], [35], [36], [37]. After the initial priming of autoreactive T cells, these cells might facilitate the activation of other self-specific CD4 T cells if both antigens are presented by the same APC. Our findings suggest that this APC would most likely be an autoreactive B cell. According to our observations, splenic B cells but not splenic DC can mediate CD4 T cell cooperation. After initial priming by DC, CD4 T cells migrate to the border of the B cell follicle where antigen-specific B cells present peptides derived from the same antigen [38], [39]. Since B cells preferentially acquire and present antigens that interact with the B cell receptor, they are ideally positioned to mediate interactions between CD4 T cells specific for peptides derived from the same, or physically linked, antigens. Sequential interactions with DC and B cells may enhance the activation and or proliferation of CD4 T cells [40]. Indeed our data would appear to reflect this phenomenon as mice given pulsed total spleen APC generate greater numbers of peptide-specific effector CD4 T cells than mice given purified B cells or DCs alone (compare Figure 2 to Figures 4 and 5). We suggest, based upon our observations on the role of OX40/OX40L interactions, that the pertinent B cell mediating the CD4 T cell cooperation in this niche is activated by the helper CD4 T cell to express OX40L, allowing it to deliver activating signals to the responding CD4 T cell, in part via OX40.

The antigen-specific nature of B cells as APC should minimize promiscuous cooperation between CD4 T cells that would lead to uncontrolled epitope spreading, which would occur if DC efficiently mediated CD4 T cell cooperation between unrelated CD4 T cells. The critical difference, besides antigen-specificity, that distinguishes B cells from DCs as APC, in mediating cooperation between CD4 T cells, is not clear. Possibilities include the differential expression of cell surface co-stimulatory molecules and/or cytokines, or the availability of these cell types in different physical locations within different zones of secondary lymphoid tissue.

It has recently become more accepted that depletion of B cells is an effective treatment of various autoimmune diseases, including some in which the damaging response appears to be predominantly due to cell-mediated autoimmunity [41]. The effectiveness of such treatment is understandable if B cells mediate interactions between autoimmune CD4 T cells that facilitate their continual interaction and activation.

In bringing together diverse observations, we have attempted to outline a framework for how B cell-mediated CD4 T cell cooperation in the activation of CD4 T cells may be involved in diverse situations such as the generation of both normal and pathogenic effector CD4 T cells and in the generation of memory CD4 T cells. Such a framework is important if the role of B cell-mediated CD4 T cell cooperation is to be exploited to increase both beneficial and undermine detrimental immune responses.
